# Flavor Characteristics, Antioxidant Activity and In Vitro Digestion Properties of Bread with Large-Leaf Yellow Tea Powder

**DOI:** 10.3390/foods13050715

**Published:** 2024-02-26

**Authors:** Gexing Zhang, Yang Zhong, Xinzhen Zhang, Yuqi Wang, Yue Sun, Xueling Li, Zhengquan Liu, Jin Liang

**Affiliations:** State Key Laboratory of Tea Plant Biology and Utilization/International Joint Laboratory on Tea Chemistry and Health Effects of Ministry of Education, Key Laboratory of Jianghuai Agricultural Product Fine Processing and Resource Utilization, Ministry of Agriculture and Rural Affairs, Anhui Engineering Research Center for High Value Utilization of Characteristic Agricultural Products, College of Tea & Food Science and Technology, Anhui Agricultural University, Hefei 230036, Chinayuesun@ahau.edu.cn (Y.S.);

**Keywords:** large-leaf yellow tea, bread, flavor, amino acid, antioxidant

## Abstract

Foods containing tea could be widely utilized due to the addition of good tea ingredients, especially large-leaf yellow tea, which is rich with a good flavor. Applying this change to bread containing tea would improve its product quality. In this research, large-leaf yellow tea bread (LYB), possessing a special flavor, was developed using ultrafine large-leaf yellow tea powder and flour as the main raw materials. The amount of ultrafine large-leaf yellow tea powder added to bread was optimized using texture, sensation, and specific volume as comprehensive evaluation indicators. At the optimal dosage, the free amino acids, volatile flavor compounds, antioxidant activity, and in vitro starch digestibility of LYB were measured. Response surface optimization experimental results showed that the comprehensive score of bread was highest when the added amount of ultrafine large-leaf yellow tea powder was 3%. In particular, compared to blank bread (BB), adding ultrafine large-leaf yellow tea powder into bread could effectively increase its amino acid composition, enhance its volatile flavor compounds, improve the antioxidant capacity, and reduce the digestibility of starch.

## 1. Introduction

Bread, as one of the economic staple foods consumed globally, is a fermented baked product typically made from wheat flour and processed through fermentation, shaping, fermentation, and baking [1]. However, bread is high in carbohydrates (mainly starch), accounting for about 50% of its dry weight [2,3]. Regular consumption might lead to cardiovascular disease, diabetes, and other illnesses. Therefore, adding functional ingredients to bread, such as large-leaf yellow tea, may have a positive impact on health [4].

Large-leaf yellow tea is a type of Chinese tea that has been slightly fermented and possesses a unique mellow flavor. It not only contains a large number of phenolic substances but also contains nutrients such as amino acids, minerals, sugars, vitamins, etc. Large-leaf yellow tea also performs functions such as regulating blood sugar, improving chronic inflammation, and preventing cardiovascular diseases and has antioxidant, anticancer, and antibacterial properties [5,6,7]. The typical processing techniques for large-leaf yellow tea include greening, smothering, and drying. Among them, special processing techniques (smothering) could help large-leaf yellow tea to produce special flavors, promote the conversion of ester-type catechins, and significantly affect primary compounds, such as theanine and tea polyphenols. Thus, a unique “potpourri” flavor is formed [8], along with three quality characteristics: yellow leaves, yellow stems, and yellow soup [9,10]. These unique flavor qualities make them more suitable as raw materials for baked foods made with tea.

Therefore, the aim of this study was to respond to the optimization of the formulation parameters of bread made with large-leaf yellow tea powder. Meanwhile, the flavor, antioxidant properties, and in vitro digestion of bread with or without large-leaf yellow tea powder were evaluated. This study aims to provide a reference value for the application of large-leaf yellow tea in bread.

## 2. Materials and Methods

### 2.1. Materials

The ingredients used were as follows: large-leaf yellow tea (Anhui Province Hold’er Zhongxiu Tea Co., Ltd., Anhui, China), flour (Weifang Kite Flour Co., Ltd., Shandong, China), edible salt (China Salt Dongxing Salt Chemical Co., Ltd., Anhui, China), xylitol (Gan Juyuan Co., Ltd., Jiangxi, China), high-activity dry yeast (Angie’s Yeast Co., Ltd., Jiangxi, China), and butter (Angie’s Yeast Co., Ltd., Jiangxi, China). ABTS (2,2-diazo-di (3-ethyl-benzothiazole-6-sulfonic acid) diammonium salt) and DPPH (1,1-diphenyl-2-trinitrophenylhydrazine) were purchased from Aladdin Reagent (Shanghai) Co., Ltd., Shanghai, China.

### 2.2. Methodologies

#### 2.2.1. Preparation of Large-Leaf Yellow Tea Powder

After crushing the large-leaf yellow tea, a ball mill was used to ultrafine crush the tea for 2 h to obtain ultrafine large-leaf yellow tea powder, which was sealed and stored at −20 °C to be kept in reserve.

#### 2.2.2. Bread Making

The process was carried out with reference to GB/T 35869-2008 and was slightly modified. Taking 100 g of flour as a base, large-leaf yellow tea powder (average particle size 25.31 μm) was substituted for flour at additive amounts of 1%, 3%, 5%, 7%, and 9%, xylitol 12%, salt 1%, dry yeast 1.5%, butter 12%, and water (93% of the water absorption measured according to the Mixolab mixing tester). The control group used plain wheat bread without the addition of large-leaf yellow tea powder. The ingredients were put into a mixer and blended until a glove film could be pulled out and were relaxed for 10 min at room temperature; then, they were deflated and plasticized, placed in a fermenter to ferment (temperature: 35 °C, relative humidity: 75%), and then taken out after 70 min and placed in an oven (190 °C) to bake for 25 min to finally obtain the bread.

#### 2.2.3. Sensory Scoring

According to GB/T 20981-2021, the scoring rules used for the sensory evaluation of bread were formulated to evaluate the smell, color, texture (softness and viscosity), and tissue. A review team of 10 members (5 men and 5 women) with more than two years of experience in food review was formed after 14 days of training. According to the sensory evaluation rules, the sensory qualities of different breads were scored one by one. Each sample was repeated three times to eliminate errors and calculate the average. The panelists were informed of the objectives of the study and their personal data were processed. An informed consent form was provided, clearly outlining the voluntary nature of participation, the right to withdraw at any time, and its confidentiality. Score sheets, data collection, and data processing were performed using Origin 2021 software.

#### 2.2.4. Determination of Bread Specific Volume

After the bread was cooled at room temperature, the volume of bread was measured using the canola substitution method [11], and the specific volume of bread (mL/g) was based on the ratio of the volume of bread to the mass.

#### 2.2.5. Bread Hardness Measurement

After cooling the bread at room temperature, the bread was subjected to the texture profiling analysis (TPA) test using a texturizer. The parameters of the texturometer were set as follows: trigger force 5 g; pre-test speed 2 mm/s; test speed 2 mm/s; post-test speed 2 mm/s; compression level 50%; and 2 presses.

#### 2.2.6. Bread Weighted Score Calculation

This detection method was based on reported literature [12]. Mainly adopting the percentage system, three indicators were selected, including bread hardness, specific volume and sensory score. A total of 10 points were assigned to the maximum value of hardness (max) and 100 points to the minimum value (min); the maximum value for specific volume and sensory scores was 100 points and the minimum value was 10 points. Then, based on the importance of each indicator, corresponding weights were assigned to calculate the comprehensive score. The specific calculation formula was as follows:

$$Y_{1}=90\times \frac{y_{1max}-y_{1}}{y_{1max}-y_{1min}}+10$$

$$Y_{2}=90\times \frac{y_{2}-y_{2min}}{y_{2max}-y_{2min}}+10$$

$$Y_{3}=90\times \frac{y_{3}-y_{3min}}{y_{3max}-y_{3min}}+10$$

Overall rating = Y1 × 0.3 + Y2 × 0.3 + Y3 × 0.4

Eq: *Y*_1_—hardness score; *y*_1_—hardness determination value; *Y*_2_—specific volume score; *y*_2_—specific volume determination value; *Y*_3_—sensory score; *y*_3_—sensory score determination value.

#### 2.2.7. Response Surface Optimization Design

The experimental optimization was mainly achieved through Box–Benhnken design. On the basis of the one-way test, three factors, namely, the amount of big-leaf yellow tea powder added (%), the amount of xylitol added (%), and the fermentation time (min), were selected for the optimization test. At the same time, three levels were determined for each factor on the basis of a single factor. The specific response surface experimental design is shown in Table 1. The comprehensive score was based on sensory and texture test values. The calculation method was as follows.

#### 2.2.8. Determination of Amino Acids in Bread

A total of 0.1 g of dry bread sample was accurately weighed, then placed into a 10 mL centrifuge tube, to which 4–10 mL of 4% yellow base salicylic acid was added, and soaked in an ultrasonic instrument for 30 min; finally, it was mixed upside down every 5 min. After standing for 10 min, 1.5 mL of the supernatant was placed into a 2 mL centrifuge tube for centrifugation (12,000 rmp/min; centrifugation for 30–40 min). Then, 1mL of the supernatant was taken, passed through a 0.22 um disposable water membrane, and placed in a sample bottle for measurement.

#### 2.2.9. Volatile Components Determination of Bread by Gas Chromatography–Ion Mobility Spectrometry (GC-IMS)

##### Sample Treatment

A total of 2 g of the sample was put in a 20 mL headspace bottle, incubated at 60 °C for 15 min, and then injected at 200 μL.

##### GC-IMS Conditions

The analysis time was 30 min. The column type was MXT-WAX, 15 mL, 0.53 mm ID, 1 um FT. The column temperature was 60 °C. The injection needle temperature was 85 °C. The IMS temperature was 45 °C. The carrier gas was high-purity N2 (99.999%) with an initial flow rate of 2 mL/min. It was incrementally increased to 1002 mL/min within 25 min.

#### 2.2.10. Antioxidant Activity Assay

##### Sample Preparation

Determined by reference method [13]. After freeze-drying and crushing the bread, 2 g of sample powder was added to 40 mL of 80% methanol solution for extraction twice. Each time, the mixture was put into a water-bath thermostatic oscillator to extract for 1 h at 200 r/min and 37 °C, and then the mixture was centrifuged at 4000 r/min for 5 min. Then, the supernatant obtained each time was combined and put into the refrigerator for use.

##### Determination of Free Radical Scavenging by DPPH

The measurement method was based on Reference [14] with appropriate modification. A 0.1 mmol/L DPPH ethanol solution was prepared and 1 mL of sample extract and 3 mL of DPPH solution were taken, mixed thoroughly, reacted under dark conditions for 30 min, and then the absorbance of the reaction solution measured at a wavelength of 517 nm using a UV spectrophotometer A. These samples were protected from light all the way. The control group used anhydrous ethanol instead of DPPH solution and sample extract, while the blank group used anhydrous ethanol instead of sample extract and DPPH solution. The absorbance was recorded as a control and a blank group, respectively. The formula for the calculation method was as follows:

$$DPPH—free\;radical\;scavenging\frac{rate}{\%}=\left(1-\frac{A_{2}-A_{1}}{A_{0}}\right)\times 100$$

where A_0_ blank is the absorbance value after the reaction of 1 mL of anhydrous methanol with 4 mL of DPPH solution; A_1_ control is the absorbance value after reaction of 1 mL extract with 4 mL anhydrous methanol; and A_2_ sample is the absorbance value after reaction of 1 mL extract with 4 mL DPPH solution.

##### Determination of ABTS Radical Scavenging Rate

The ABTS was determined by the method of reference [15]. A 7 mmol/L ABTS solution and 2.4 mmol/L potassium persulfate solution were prepared, respectively, mixed at 1:1, and reacted for 16 h under the condition of light avoidance; then, an appropriate amount of the reaction solution was taken and diluted with anhydrous ethanol so as to make the absorbance of the solution at 734 nm 0.70 (±0.02), and then the ABTS working solution was obtained. The working solution of ABTS was obtained by mixing the sample extract with the working solution of ABTS in the ratio of 1:8, and the reaction was carried out for 6 min under the protection of light. The absorbance of the solution was measured at 734 nm, which was labeled as the A sample. The control group was treated with anhydrous ethanol instead of ABTS working solution and sample extract. In the blank group, anhydrous ethanol was used to replace the sample extract with ABTS working solution, and the absorbance was recorded as a control and blank, respectively. The calculation formula was as follows:

$$\mathrm{ABTS}\;\mathrm{free}\;\mathrm{radical}\;\mathrm{scavenging}\;\mathrm{rate}/\%=(1-\frac{A_{2}-A_{1}}{A_{0}})\times 100$$

where A_0_ blank is the absorbance value after the reaction of 1 mL of anhydrous methanol with 4 mL of DPPH solution; A_1_ control is the absorbance value after reaction of 1 mL extract with 4 mL anhydrous methanol; and A_2_ sample is the absorbance value after reaction of 1 mL extract with 4 mL DPPH solution.

##### In Vitro Digestion Assay

In vitro digestion was determined based on relevant literature with slight modifications [16]. A total of 200 mg of sample powder was mixed with 5 mL of 0.2 mol/L pH 5.2 sodium acetate buffer solution, shaken thoroughly, and placed in a boiling water bath for 10 min, then cooled to 37 ° C in the water bath. Then, 16 mL of 1.42 mg/mL porcine pancreatic α-amylase solution and 4 mL of 0.2 mg/mL saccharase solution were mixed into enzyme solution and put into a water bath at 37 °C for 6 min. The enzyme solution was mixed with the sample solution and shaken well and then put into a 37 °C thermostatic shaking water bath (160 r/min) to start the enzyme digestion. The time arrangement was very accurate at hydrolysis for 0, 10, 20, 30, 60, 90, 120, and 180 minutes, respectively. A total of 1 mL of the sample was taken and 4 mL of anhydrous ethanol was added to the sample solution and shaken well and then centrifuged at a speed of 4000 r/min for 5 min. A total of 1 mL of the supernatant was taken into a 25 mL glass stoppered tube and then 1 mL of water and 1.5 mL of DNS reagent were added in turn (configured one week in advance), and it was placed into cold water immediately after a boiling water bath for 5 min. After 5 min in a boiling water bath, it was put into cold water and immediately cooled down to 25 mL. Finally, the absorbance was measured at 510 nm and glucose was used as the standard to measure the absorbance and plot the standard curve. The glucose content and starch hydrolysis rate were calculated according to the DNS standard curve.

$$RDS(\%)=\frac{(G_{20}-FG)\times 0.9}{TS}$$

$$SDS(\%)=\frac{{(G}_{120}-G_{20})\times 0.9}{TS}$$

$$RS(\%)=\frac{\left[TS-(RDS+SDS)\right]}{TS}$$

where *G*_20_ is glucose content (mg) after 20 min of hydrolysis; *G*_120_ is glucose content after 120 min of hydrolysis (mg); *FG* is free glucose content in sample (mg); and *TS* is total starch content in samples.

The hydrolysis curve was fitted using the first-stage reaction equation:

C = C∞ (1 − e − kt)

where C is starch hydrolysis rate at different times (%); t is digestion reaction time (min); k is kinetic constant of the first-stage reaction; and C∞ represents the equilibrium value of starch hydrolysis rate (%) after 180 min of hydrolysis. The starch hydrolysis curve was fitted to derive the C∞ and k values. The hydrolysis index (HI) was obtained from the following equation:

$$HI(\%)=\frac{Area\;under\;the\;sample\;curve\;(0-189\;min\;)}{Area\;under\;the\;white\;bread\;digestion\;curve\;(0-180\;min\;)}\times 100$$

The area under the hydrolysis curve is given by the following equation:

$$AUC=C_{\infty }(t_{\infty }-t_{0})-(C_{\infty }/k)[1-exp[-k(t_{\infty }-t_{0})]]$$

where t_∞_ and t_0_ represent the final digestion time (180 min) and initial time (0 min), respectively, and k is a kinetic constant.

White bread was used as a standard and its glycemic index (GI) was set at 100; there was a high correlation between HI and GI (r = 0.894), and the formula for predicting GI from HI was as follows:

eGI=0.549HI+39.71

##### Statistical Analysis

All data were replicated at least 3 times, data were processed using Office software, SPSS 26.0 software was used to analyze the data for ANOVA significance of difference, with *p* < 0.05 indicating significant difference, and Origin 2019b software was used for graphing. PCA plots and PLS-DA models were plotted using SIMCA (version 14.1) and alignment tests were performed to determine the accuracy of the models.

## 3. Results and Analysis

### 3.1. Response Surface Analysis

The response surface values were analyzed using Design Expert 8.0.6 software and the formula:

Y = 71.34 − 0.059A − 1.08B − 0.29C − 1.38AB + 2.56AC + 2.31BC − 4.59A2 − 7.53B2

where Y is the composite score, A is the amount of xylitol added (%), B is the amount of large-leaf yellow tea powder added (%), and C is fermentation time (min).

As shown in Table 2, the adjusted R2 was higher than 90% and the model fit well. *p*-value was less than 0.01, which indicated that the model was highly significant. The effect of each factor on the composite score and the interaction between the factors could be obtained from the response surface data. The model showed that the optimal process parameters for bread were 2.75% addition of tea powder, 11.99% addition of xylitol, 69.5 min of fermentation time, and a comprehensive score of 71.46 for bread. Based on practical operations, the optimized parameters were obtained as 3% addition of tea powder, 12% addition of xylitol, and a fermentation time of 70 min. Three parallel experiments were conducted and the comprehensive score was measured to be 71.75 ± 0.63, which was close to the predicted value.

This indicated that the model was reliable and could be used for the application of large-leaf yellow tea bread.

### 3.2. Basic Components

In Table 3, compared to the blank bread, the value of protein, fat, dietary fiber, ash, and starch in large-leaf yellow tea bread increased by 8.1%, 10.4%, 18.7%, 48.6%, and 27.2%, respectively. Especially, the maximum increased in ash content. Similar reports could also be expressed as mineral content [17]. In addition, the report showed that large-leaf yellow tea itself contained a large amount of minerals, such as iron, copper, and zinc [18]. The comprehensive analysis concluded that the nutrient content of large-leaf yellow tea bread was superior to that of blank bread.

### 3.3. Amino Acid Analysis of Bread

The types and contents of free amino acids in different breads are shown in Table 4. It could be seen that the amino acid contents of large-leaf yellow tea bread (LYB) and blank bread (BB) were different. Meanwhile, the types of amino acids were also different. Both types of bread contained 15 flavor-free amino acids, including 7 and 6 essential amino acids and 8 and 9 non-essential amino acids, respectively. It was known that the freshness amino acids were mainly determined by glutamic acid and aspartic acid, and glutamic acid was the most important amino acid in freshness [19]. It could effectively promote liver metabolism. LYB was rich in six types of fresh amino acids, which was 5.34% higher than BB. Especially, aspartic acid was relatively high. In addition, LYB also contained a special flavorful amino acid theanine, which gave bread a special freshness quality. Generally, methionine produced an unpleasant aroma. Compared with the control group, LYB lacked one amino acid, methionine. It was speculated that the reason for its occurrence was that, during the high-temperature baking process, methionine reacted with substances in large-leaf yellow tea and converted them into alcoholic amino acids. It was also possible that methionine was further degraded in the Strecker reaction to produce ethylene [20]. Therefore, the addition of large-leaf yellow tea was more beneficial to improve the flavor quality of bread.

TAV was the ratio of the concentration of the flavor-presenting substance in the sample to its taste threshold, which could reflect the taste intensity of the flavor-presenting substance of the sample. When TAV > 1, it represented that the amino acid contributed to presenting the flavor. The bigger the value of TAV, the better the effect of presenting flavor. When TAV < 1, it represented that the amino acid had no contribution to the flavor presenting. The smaller the value, the worse the effect of presenting flavor. In Table 4, it could be seen that there were two types of amino acids in BB that contribute to the flavor, namely glutamic acid and alanine. Among them, glutamic acid had the highest TAV value, indicating that glutamic acid could provide strong freshness and significantly contribute to the flavor of BB. In contrast, there were three fresh amino acids that contributed to flavor presentation in LYB, namely glutamic acid, alanine, and theanine, all with higher TAV values than that of BB. Meanwhile, the unique one, theanine, had the highest TAV value, which acted with other fresh amino acids to give a better fresh flavor. The TAV values of bitter amino acids, sweet amino acids, and aromatic amino acids of both breads were less than 1, which did not contribute to the bread flavor presentation. The order of ranking of amino acid species contributing to flavor presentation for both breads was summarized as LYB (3) > BB (2).

### 3.4. GC-IMS Analysis

**Figure 1:**
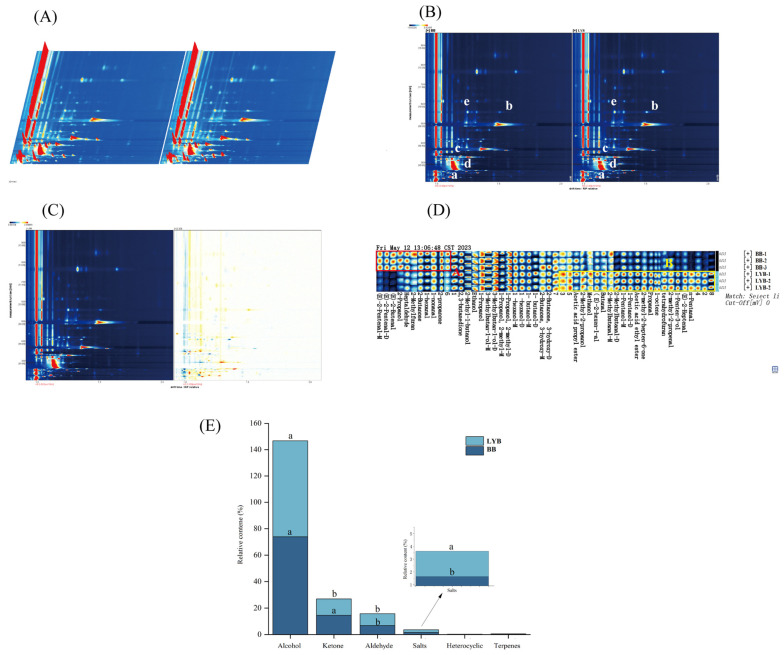
Three-dimensional spectrum of volatile matter composition in the sample (**A**), volatile substance composition spectrum in the sample (**B**), comparison and difference spectrum of volatile substance composition in the sample (there are differences at the marked positions “a”, “b”, “c”, “b”, “c”, “d”, and “e”) (**C**), sample gallery plot fingerprint spectrum (**D**), and relative content of various bread compounds (**E**).

The three-dimensional spectra of volatile matter compositions of the two breads are shown in Figure 1A. BB and LYB visualized similarly but with different intensities for different breads. It indicated that the distribution of volatile substances was different for the two breads. The ion migration time and reaction peak positions could be normalized to distinguish the different volatile distributions of BB and HTB. Figure 1B shows the two-dimensional spectra of the two kinds of bread; the vertical co-ordinate represents the retention time (s) of the gas chromatogram and the horizontal co-ordinate represents the ion migration time (normalization). The reactive ion peak (RIP) was located in the red vertical line at the horizontal co-ordinate 1.0. Each point on both sides of the RIP represented a kind of volatile organic compound. It could be visualized based on the presence or absence of peaks (color dots) or the color depth of the peaks (color dots). The presence or absence of the peaks (color dots) or the color shades could visualize the differences in components and concentrations between samples (white color corresponds to the low concentration of volatiles and red color corresponds to high concentration) [21]. From Figure 1, it could be concluded that the volatiles were mainly concentrated in the drift time of 0–15 s and the retention time of 0–500 s. 

In order to highlight more significantly the difference between the flavor substances of the two breads, one bread sample was selected as a reference. If the other sample had the same volatile substance, the background was white after removing the same substance. The red color represented that the substance had a higher concentration in that sample than in the reference sample, while the blue color represented a lower concentration than that in the reference sample [22]. As shown in Figure 1C, more red dots were found in the retention time range of HTB compared to that of BB. This result suggested that breads with the addition of large-leaf yellow tea powder would result in the appearance of signals or an increase in the intensity of signals for some of the compounds, which would lead to a significant increase in the flavor content of LYB.

Based on the analysis of Figure 1D and Table 5, a total of 42 volatile compounds were identified from the two samples. These aroma active substances were mainly formed due to the hydrolysis of glycosides, degradation of carotenoids, degradation of lipids, and Maillard reaction during processing [23]. Eight pairs of the 42 volatile compounds were monomer dimers or polymers. A total of 19 alcohols, 12 aldehydes, 2 esters, 6 ketones, 2 heterocycles, and 1 terpene were identified. The volatile organic compound (VOC) fingerprints of different breads revealed that the flavor substance content of bread samples with the addition of large-leaf yellow tea powder changed significantly. From the figure, it could be seen that the content of trans 2-pentenol, (E)-2-butene, 2-propanol, heptanal, 2-methylfuran, 2-butanone, 1-hexanal, nonanal, and 2-acetone in BB was relatively high, as shown in the selected region A in the figure. This indicated that the content of volatile compounds in BB was higher than that in LYB, while the content of volatile compounds in region B was higher. Among them, the content of volatile compounds in LYB was higher than that in BB, which could be used as a characteristic substance for identifying LYB.

The aroma of bread was derived from more than 300 analytes. It was influenced by the use of raw materials and by dough fermentation, lipid oxidation, enzymatic reactions, reactions in microbial cells, and baking in bread production [24,25]. From the data in Figure 1E, it could be seen that the highest relative content of volatile components in BB and LYB were alcohols, which were important constituents of bread flavor substances. Alcohols were the main volatile substances in the samples. They played a crucial role in the aroma building blocks of bread. According to Table 5, they mainly included 1-hexanol, 1-pentanol, 1-butanol, 3-methyl-1-butanol, 1-propanol, ethanol, etc. Among these alcohol compounds, ethanol had the highest relative content and also the highest relative content among all volatile components. This was because the metabolism of yeast in bread could convert fermentable sugars into ethanol, most of which evaporates during the baking process. The remaining could participate in secondary fermentation reactions, such as glycolysis of pyruvate, ultimately producing short-chain alcohols, short-chain fatty acids, carbonyl compounds, and esters. The amino acids present in the secondary fermentation were gradually absorbed by the yeast cells throughout the fermentation process and after transamination reaction. They were converted to heterohydric aldehydes and finally reduced to heterohydric alcohols or oxidized to heterohydric alcohols, which resulted in richer flavor of the bread [26]. 1-Pentanol, 1-butanol, and 1-penten-3-ol had a balsamic, fruity, buttery, boozy, and sweet flavor. Their relative content in LYB was significantly higher than that in BB. 3-methyl-1-butanol was the most important fermentation aroma compound due to its high odor active value (OAV), high flavor dilution factor (FD), and positive correlation with wheat bread aroma. It mainly presented alcoholic, fruit, almond, and burnt flavors, which would increase consumer acceptance [27,28]. Especially, 3-methyl-1-butanol was converted from leucine, and the value in LYB was higher than that in BB, which corresponded to the amino acid results, indicating that the addition of yellow tea powder was beneficial for increasing the aroma of bread.

From Figure 1E, it could be seen that LYB and BB had similar types of volatile compounds. However, the amount of each volatile compound was different. Aldehydes could be generated in two ways. One could be generated by the Strecker process of alcohols as well as amino acids and the other was formed by the degradation of fatty acids through the Ehrlich pathway [29]. The relative content of aldehydes was significantly higher in LYB than that in BB. 2-methylbutyraldehyde had a malt and almond aroma [30] and was 2.9 times higher relatively in LYB. The propionaldehyde had a malt aroma and was 2 times higher relatively in LYB; E-2-heptenal had a fat and almond aroma and was 1.8 times higher relatively in LYB; 2-methacrylaldehyde was 2 times higher relatively in LYB. N-pentanal had a fruity odor, the relative amount of which in LYB was 7.7 times that of BB. These aldehydes had a low odor threshold and contribute to the aroma profile of bread [31].

Esters were synthesized through the esterification of alcohols and free fatty acids produced by fat oxidation. They were common volatile compounds. However, due to their lower odor threshold, they did not contribute significantly to the aroma of bread [32]. Ethyl acetate was a volatile compound positively correlated with bread aroma. The relative content of ethyl acetate in LYB was significantly higher than that in BB. It could give bread a better fruity and floral flavor [33]. Most ketones were produced through the degradation of unsaturated fatty acids or amino acids, and there was not much difference in ketone content between the two types of bread. Among them, 2,3-butanedione and 3-hydroxy-2-butanone were positively correlated with the aroma of bread. There was not much difference between 3-hydroxy-2-butanone compared to BB, and the content of 2,3-butanedione in LYB was lower. It had a butterscotch and caramelized odor [34]. The two types of bread only produced two heterocyclic compounds and one terpene, tetrahydrofuran, 2-methylfuran, and 1-octene, with little difference in content, which was considered a difference in sugar types. It affected the results of the Melad reaction, when compared with a similar report [35].

**Figure 2:**
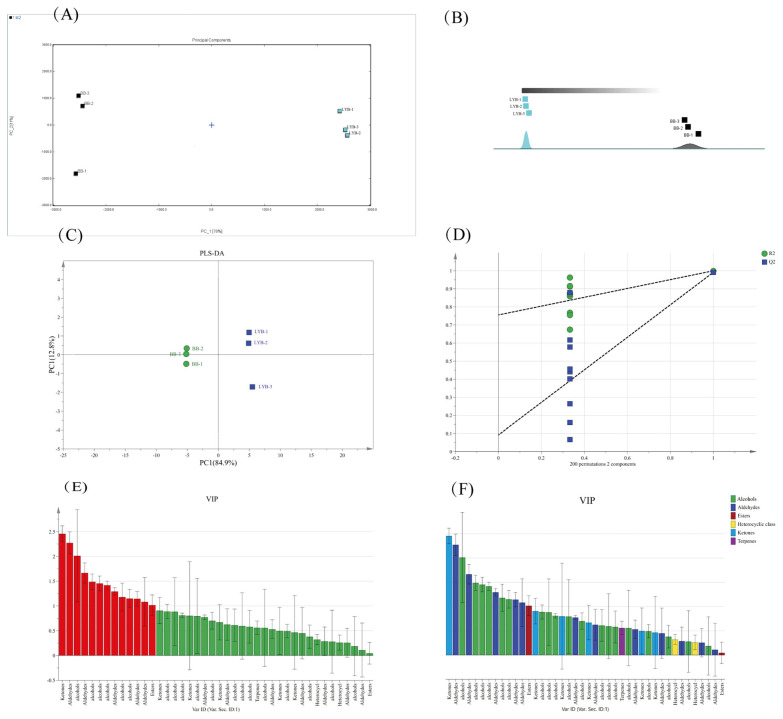
Bread-to-bread PCA analysis (**A**), Euclidean distance plot between bread (**B**), OPLS-DA score of bread sample (**C**), 200-permutation test of OPLS-DA (**D**), VIP plot of OPLS-DA (**E**,**F**).

In order to make it easier to see the differences in volatiles between the two breads, a principal component analysis (PCA) plot was produced and the results are shown in Figure 2A. The principal component analysis plot was used as a multivariate data processing technique to identify complex and hard-to-find variables in order to assess the ability of differences and regularities between samples [36,37]. The horizontal and vertical co-ordinates were the first principal component (PC1) and the second principal component (PC2), respectively. The sum of the cumulative contributions of the two principal components after dimensionality reduction was 89% (PC1 is 78%; PC2 is 11%). After feature compression, relatively complete information was still retained. It could better characterize the feature differences of the original variables and represent most of the information of the compound. From the PCA plot, it could be seen that the two kinds of breads were distributed in different areas of the plot. There was a clear separation between the samples on principal component 1, with a close distance and good parallelism within the sample group. The samples were farther away from each other, with obvious characteristic differences. It indicated that the volatile compounds of the bread had changed significantly after the addition of large-leaf yellow tea powder. Combined with the Euclidean distance between the samples in Figure 2B, it could also be seen that the distance between the samples was significantly larger than the distance within the group. The difference between the groups was significant, which could be used to directly cluster the two types of samples by the distance.

The orthogonal partial least squares discriminant analysis (OPLS-DA) was an improved partial least squares discriminant analysis (PLS-DA) method. Compared with other methods, it was easier to exclude independent variables that were not related to classification. The OPLS-DA model had been widely used for food traceability by screening the characteristic variables of the sample [38]. After passing 200 cross-tests, the horizontal co-ordinate represented the similarity with the original model. Where R2X and R2Y denote the explanatory rate of the constructed model for the X and Y matrices, respectively. Q2 denoted the predictive power of the model. Theoretically, the closer the R2 and Q2 values were to 1, the better the model. The lower it was, the worse the fitting accuracy of the model. From Figure 2D, Q2 was 0.983, R2X was 0.855, R2Y was 0.996, and the *p*-values of Q2 and R2 were less than 0.01. This indicated that the validity criteria of the original OPLS model were satisfied. These results showed a significant difference between BB and LYB pairs. The variable importance for the projection (VIP) plots obtained after analyzing the OPLS-DA model could determine the contribution of each variable to effectively distinguish between category samples. The variables with VIP > 1 were considered to have the greatest impact on the model [39]. It was generally accepted that variables with VIP values > 1 were significantly different between categories and played an important role in categorization. A total of 12 compounds with VIP values greater than 1 can be seen in Figure 2E. Five alcohols, one aldehyde, one ketone, and one ester can be specifically seen in Figure 2F.

### 3.5. Antioxidant Properties of Bread

**Figure 3:**
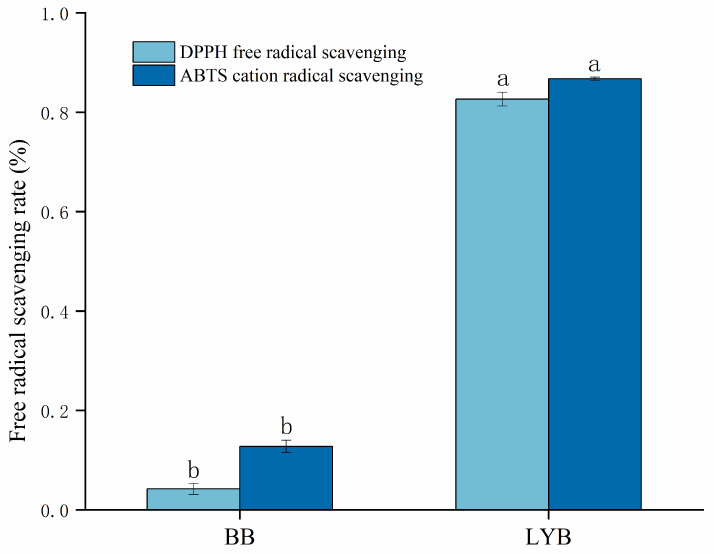
Antioxidant activity of the two breads. The average values ± SD in individual columns marked with the same superscripts do not differ statistically significantly (*p* < 0.05).

As shown in Figure 3, the antioxidant capacity of LYB was significantly enhanced compared to that of BB. Large-leaf yellow tea contained a large amount of catechins and phenolics [40], which could enhance the antioxidant capacity of bread. In addition, large-leaf yellow tea contained a large amount of dietary fiber. It could scavenge the function of free radicals. Moreover, it might be involved in the formation of melanoidins, which were the browning pigments produced during the baking process and also had antioxidant properties [41]. Therefore, the addition of large-leaf yellow tea powder could effectively improve the antioxidant capacity of bread.

### 3.6. In Vitro Digestive Analysis

The starch portion rapidly digested and absorbed in the small intestine was called rapidly digestible starch (RDS). For chronic digestive starch (SDS), the starch portion was digested slowly in the small intestine. The part of the starch that could not be digested by α-amylase and glucosidase after 120 min was called resistant starch (RS). Starch digestibility was affected by a number of factors, such as crystallinity, particle size, ratio of straight-chain starch to linear-chain starch, and source [42,43]. In Table 6, the RDS and SDS contents of LYB were significantly lower than that of BB, whereas the RS content was significantly higher than that of BB. It indicated that the addition of large-leaf yellow tea powder was beneficial in inhibiting starch digestion. The decreasing contents of RDS and SDS and increasing content of RS indicated that starch digestion in bread was inhibited. The decrease in RDS and SDS content might be due to the high content of tea polyphenols in large-leaf yellow tea, which contained a large number of hydroxyl groups in its molecular structure. It could bind proteins and starch through noncovalent interactions (hydrogen bonding, electrostatic, hydrophobic interactions, van der Waals forces, etc.), thereby affecting the digestion rate of bread [44,45]. In addition, the amylose formed complex complexes with tea polyphenols through hydrophobic interactions. This structure was not easily hydrolyzed during digestion. The amino acids at the binding site of digestive enzymes could form a stable conjugated mode with the hydroxyl groups of tea polyphenols through hydrogen bonding [46].

**Figure 4:**
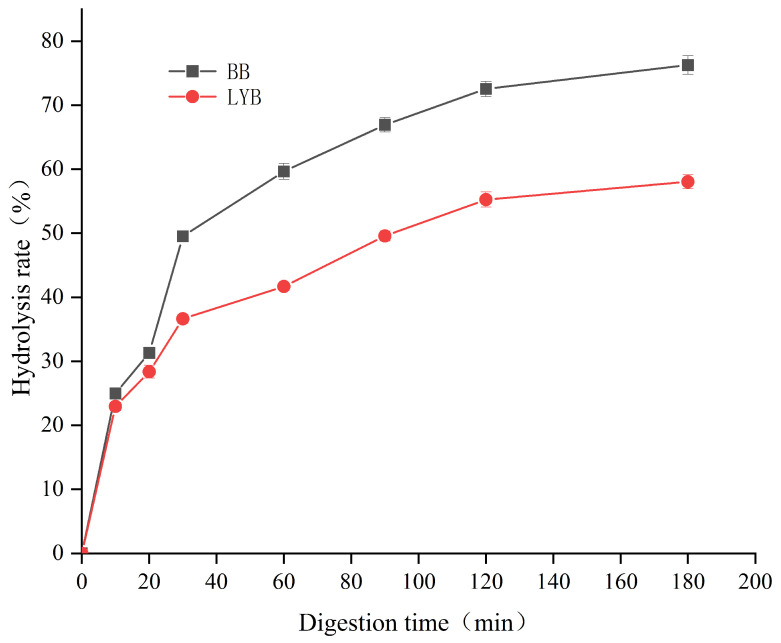
In vitro digestion profiles of different breads.

In Figure 4, under the action of enzymes, the starch digestibility of the two types of bread showed a change from fast to slow, increasing rapidly from 0 to 40 min and then decreasing after 60 min. It could be seen that the digestion level of LYB was lower than that of the control group without tea powder. This result indicated that the addition of large-leaf yellow tea powder could reduce the degree of starch digestion. It might be because large-leaf yellow tea contained dietary fiber, which was difficult to digest, and starch could interact with dietary fiber to encapsulate or form a structure that inhibited enzyme hydrolysis, reduced enzyme feasibility, and led to reduced starch digestion [47]. Previous studies had shown that dietary fiber could adsorb glucose and inhibit it. α-Amylase was used to inhibit starch digestion rate [48].

After fitting the starch digestibility of two types of bread with a first-order equation, the results are shown in Table 7. Generally speaking, low levels of RDS would reduce the values of C∞ and in vitro glycemic index (eGI) [49], corresponding to the data in Table 4. According to the results, compared with BB, the C∞ value of LYB significantly decreased. While the K value did not show significant changes. This result indicated that the presence of large-leaf yellow tea powder not only reduced the degree of starch digestion but also lowered the rate of starch digestion. The reduction in HI and eGI values might be due to the active substances contained in large-leaf tea having a hypoglycemic effect, thereby lowering the value of eGI.

## 4. Conclusions

This study mainly used large-leaf yellow tea powder as the raw material to explore its application characteristics in bread. Response surface optimization was used to obtain the optimal addition amount of tea powder. Meanwhile, the flavor compounds, amino acids, and antioxidant activity of bread with or without large-leaf yellow tea powder were compared. Response surface optimization results displayed that the addition amount of large-leaf yellow tea powder was 3%. GC-IMS analysis results showed that a total of 42 volatile compounds were identified in bread with large-leaf yellow tea powder, including 19 alcohols, 12 aldehydes, 2 esters, 6 ketones, 2 heterocycles, and 1 terpenoid. In addition, the volatile compounds of the bread changed significantly after the addition of large-leaf yellow tea powder. There were as many as 12 compounds with VIP values greater than 1. Meanwhile, the addition of large-leaf yellow tea powder in bread not only reduced the degree of starch digestion but also reduced the rate of starch digestion. In conclusion, the addition of large-leaf yellow tea powder was beneficial to improve the quality and enhance the flavor of bread.

## Figures and Tables

**Table 1:** Composition and responses in Box–Behnken Design.

Batch	Factor			Hardness		Specific Volume		Sensory Score	Response
	LYT (%)	XA (%)	FT (min)	x1	X1	X2	X2	X3	
1	1	9	70	1235.58	43.51	2.19	47.81	79.63	59.24
2	1	15	70	1524.37	57.71	2.39	50.10	77.75	63.44
3	5	9	70	1739.41	41.56	1.98	53.80	72.88	57.76
4	5	15	70	1424.29	42.99	2.54	46.64	73.88	56.44
5	3	9	60	1059.43	54.68	2.12	52.18	81.75	64.76
6	3	15	60	1810.54	56.35	2.34	40.57	72.25	57.97
7	3	9	80	889.14	52.02	2.49	44.59	73.88	58.53
8	3	15	80	1293.43	51.08	2.68	48.66	80.13	61.97
9	1	12	60	1291.51	52.64	2.62	48.51	78.38	61.69
10	5	12	60	1593.64	45.03	2.21	42.52	69.63	54.12
11	1	12	80	1274.40	46.68	2.45	42.69	75.50	57.01
12	5	12	80	1396.89	48.79	1.81	57.29	67.13	58.67
13	3	12	70	1025.48	72.45	2.80	59.63	74.00	69.22
14	3	12	70	935.39	69.47	2.62	64.32	78.75	71.69
15	3	12	70	1123.54	75.40	2.45	62.53	75.63	71.63
16	3	12	70	1056.53	74.30	2.32	59.53	79.65	72.01
17	3	12	70	998.35	71.50	2.57	62.30	79.90	72.15

LYT: large-leaf yellow tea powder addition, XA: xylitol addition, FT: fermentation time.

**Table 2:** Analysis of variance of the experimental factors on the response variable.

Source	SS	Df	MS	F-Value	*p*-Value	Significance
Model	610.58	9	67.84	39.46	<0.0001	**
A	0.028	1	0.028	0.016	0.9027	
B	25.88	1	25.88	15.06	0.0061	**
C	0.7	1	0.7	0.4	0.5448	
AB	7.62	1	7.62	4.43	0.0733	
AC	26.16	1	26.16	15.22	0.0059	**
BC	21.3	1	21.3	12.39	0.0097	**
A^2^	88.8	1	88.8	51.65	0.0002	**
B^2^	238.58	1	238.58	138.77	<0.0001	**
C^2^	148.56	1	148.56	86.41	<0.0001	**
Residual	12.03	7	1.72			
Lack of Fit	6.23	3	2.08	1.43	0.3582	
Pure Error	5.81	4	1.45			
Cor Total	622.62	16				

SS, sum of squares; df, degree of freedom; MS, mean square. ** Difference is highly significant.

**Table 3:** Basic composition of bread.

Form	Carbohydrate (g/100 g)	Fat (g/100 g)	Dietary Fiber (g/100 g)	Ash (g/100 g)	Farina (g/100 g)
BB	7.32 ± 0.02 ^b^	5.00 ± 0.10 ^b^	4.82 ± 0.02 ^b^	0.74 ± 0.01 ^b^	35.3 ± 0.10 ^b^
LYB	7.91 ± 0.02 ^a^	5.52 ± 0.02 ^a^	5.72 ± 0.03 ^a^	1.10 ± 0.10 ^a^	44.9 ± 0.20 ^a^

^a^ and ^b^ all values are means of triplicate determinations ± SD. Means with different letters in the columns are significantly different (*p* < 0.05).

**Table 4:** Results of amino acids contained in two bread samples.

Form	Amino Acid Name	Taste Threshold	BB			LYB		
			Concentration (mg/100 g)	Mass Fraction (%)	TAV	Concentration (mg/100 g)	Mass Fraction (%)	TAV
Fresh Amino Acids	Asp	100	56.34 ± 0.28 ^b^	13.06	0.56	65.78 ± 0.20 ^a^	13.77	0.66
	Glu	30	143.67 ± 0.33 ^b^	33.31	4.79	153.50 ± 0.70 ^a^	32.12	5.12
	Gly	130	14.34 ± 0.10 ^a^	3.32	0.11	8.67 ± 0.07 ^b^	1.81	0.07
	Ala	60	71.10 ± 0.17 ^b^	16.48	1.19	74.61 ± 0.01 ^a^	15.61	1.24
	Lys	50	15.01 ± 0.07 ^a^	3.48	0.3	14.12 ± 0.26 ^b^	2.95	0.28
	Theanine	6	0 ^b^	0	0	41.70 ± 0.36 ^a^	8.73	6.95
Sum			300.47 ± 0.40	69.66		358.38 ± 0.21	75	
Bitter amino acids	Arg	50	32.19 ± 0.51 ^b^	7.46	0.64	36.07 ± 2.23 ^a^	7.55	0.72
	Val	40	23.71 ± 0.17 ^a^	5.5	0.59	16.12 ± 0.13 ^b^	3.37	0.4
	Met	30	4.21 ± 0.19 ^a^	0.98	0.14	0 ^b^	0	0
	Ile	90	8.36 ± 0.03 ^a^	1.94	0.09	8.04 ± 0.16 ^b^	1.68	0.09
	Leu	190	13.48 ± 0.01 ^b^	3.59	0.08	15.52 ± 0.40 ^a^	2.83	0.07
Sum			81.96 ± 0.50	19.46		75.76 ± 2.66	15.44	
Sweet Amino Acids	Ser	150	15.60 ± 0.16 ^b^	3.62	0.1	16.29 ± 0.11 ^a^	3.41	0.11
	His	20	0.92 ± 0.01 ^a^	0.21	0.05	0.85 ± 0.01 ^b^	0.18	0.04
	Thr	260	9.92 ± 0.02 ^a^	2.3	0.04	9.89 ± 0.14 ^a^	2.07	0.04
Sum			26.44 ± 0.14	6.13		27.04 ± 0.03	5.66	0.19
aromatic amino acid	Tyr	260	10.84 ± 0.40 ^a^	2.51	0.04	9.34 ± 0.07 ^b^	1.95	0.04
	Phe	90	9.66 ± 0.13 ^a^	2.24	0.11	9.33 ± 0.18 ^a^	1.95	0.1
Sum			20.50 ± 0.26	4.75		18.67 ± 0.11	3.91	

^a^ and ^b^ all values are means of triplicate determinations ± SD. Means with different letters within a row differ significantly (*p* < 0.05).

**Table 5:** Comparison of volatile substances in different bread samples.

Form	Number	GAS	Formula	RI ^a^	Rt ^b^	Dt ^c^	Relative Content %		*p* Value
							BB	LYB	
Alcohol	1-hexanol-M	C111273	C_6_H_14_O	1367.6	761.578	1.32897	4.62	4.16	<0.001
	1-hexanol-D	C111273	C_6_H_14_O	1368.5	763.562	1.64463	1.75	1.78	0.598
	1-Pentanol-M	C71410	C_5_H_12_O	1261.3	557.248	1.2566	2.04	2.48	<0.001
	1-Pentanol-D	C71410	C_5_H12O	1262.2	558.571	1.51375	0.78	1.5	<0.001
	3-Methylbutan-1-ol-M	C123513	C_5_H_12_O	1215.6	493.28	1.24842	1.28	1.36	0.013
	3-Methylbutan-1-ol-D	C123513	C_5_H_12_O	1217	495.08	1.49074	11.22	11.26	0.175
	2-Methyl-1-butanol	C137326	C_5_H_12_O	1216.1	493.892	1.23322	0.83	0.77	0.024
	1-butanol-M	C71363	C_4_H_10_O	1153	419.057	1.18338	1.23	1.17	0.411
	1-butanol-D	C71363	C_4_H_10_O	1153	419.057	1.38245	0.8	1.07	0.078
	(E)-2-Pentenal-M	C1576870	C_5_H_8_O	1143.7	409.141	1.10791	0.34	0.13	<0.001
	(E)-2-Pentenal-D	C1576870	C_5_H_8_O	1143.3	408.728	1.36386	0.81	0.15	<0.001
	1-Penten-3-ol	C616251	C_5_H_10_O	1169.2	436.823	0.94494	0.41	1.1	<0.001
	1-Propanol, 2-methyl-M	C78831	C_4_H_10_O	1103.6	369.161	1.17157	1.4	1.24	0.001
	1-Propanol, 2-methyl-D	C78831	C_4_H_10_O	1105.2	370.716	1.36167	5.83	5.52	0.048
	1-Propanol	C71238	C_3_H_8_O	1049.5	327.327	1.25457	3.18	3.02	0.125
	2-Methyl-2-propanol	C75650	C_4_H_10_O	926	260.059	1.32396	0.63	0.77	0.013
	Ethanol	C64175	C_2_H_6_O	950.6	270.668	1.13899	36.14	34.71	0.010
	Methanol	C67561	CH_4_O	912.7	254.506	0.98512	0.33	0.59	<0.001
	2-Propanol	C67630	C_3_H_8_O	927.3	260.641	1.21717	0.2	0.12	0.002
Aldehyde	1-nonanal	C124196	C_9_H_18_O	1401.8	845.559	1.47987	1.11	1.02	0.113
	(E)-2-Heptenal	C18829555	C_7_H_12_O	1331.1	680.904	1.25814	0.55	0.98	<0.001
	Heptaldehyde	C111717	C_7_H_14_O	1193.8	465.332	1.33323	0.48	0.38	0.003
	(E)-2-hexen-1-al	C6728263	C_6_H_10_O	1227.2	508.714	1.18338	0.23	0.26	0.113
	1-hexanal	C66251	C_6_H_12_O	1096.1	362.21	1.56592	1.59	1.18	0.006
	(E)-2-Butenal	C123739	C_4_H_6_O	1059.7	334.654	1.20485	0.64	0.09	<0.001
	2-Methylbutanal-M	C96173	C_5_H_10_O	921.9	258.375	1.15718	0.2	0.16	0.084
	2-Methylbutanal-D	C96173	C_5_H_10_O	922.4	258.556	1.40197	0.66	2.34	<0.001
	2-methyl-2-propenal	C78853	C_4_H_6_O	887	244.165	1.22265	0.14	0.28	0.011
	Propanal	C123386	C_3_H_6_O	820.6	219.249	1.14364	0.94	1.84	<0.001
	N-Pentanal	C110623	C_5_H_10_O	997.9	292.513	1.43005	0.03	0.23	<0.001
	Butanal	C123728	C_4_H_8_O	886.9	244.113	1.11772	0.21	0.23	0.673
Salts	Acetic acid ethyl ester	C141786	C_4_H_8_O_2_	894.1	246.957	1.33916	1.55	1.89	<0.001
	Acetic acid propyl ester	C109604	C_5_H_10_O_2_	987.5	287.337	1.48099	0.1	0.09	0.817
Ketone	2-methyl-2-hepten-6-one	C110930	C_8_H_14_O	1347.2	715.29	1.18115	0.47	0.75	0.001
	2-Butanone, 3-hydroxy-M	C513860	C_4_H_8_O_2_	1297.4	614.117	1.06104	0.82	0.72	0.041
	2-Butanone, 3-hydroxy-D	C513860	C_4_H_8_O_2_	1296.3	612.133	1.33359	0.64	0.76	0.231
	2,3-butanedione	C431038	C_4_H_6_O_2_	1025.7	310.761	1.18122	4.43	4.11	0.017
	2-Butanone	C78933	C_4_H_8_O	911	253.83	1.24697	0.99	0.83	0.013
	2-propanone	C67641	C_3_H_6_O	839.1	225.907	1.11628	7.19	5.22	<0.001
Heterocyclic	Tetrahydrofuran	C109999	C_4_H_8_O	846.4	228.608	1.06567	0.09	0.12	0.016
	2-Methylfuran	C534225	C_5_H_6_O	864.1	235.231	0.98536	0.17	0.13	0.001
Terpenes	1-octene	C111660	C_8_H_16_	846.8	228.735	1.17145	0.11	0.32	<0.001

^a^ Represents the retention index (RI) calculated using n-ketones C4–C9 as external standard on FS-SE-54-CB column. ^b^ Represents the retention time (RT) in the capillary GC column. ^c^ Represents the drift time (Dt) in the drift tube.

**Table 6:** Classification of in vitro digestion of starch for different breads.

	RDS (%)	SDS (%)	RS (%)
BB	20.38 ± 2.23 ^a^	37.09 ± 0.93 ^a^	42.53 ± 1.72 ^b^
LYB	11.34 ± 0.68 ^b^	24.20 ± 0.26 ^b^	64.46 ± 0.70 ^a^

^a^ and ^b^, all values are means of triplicate determinations ± SD. Means within columns with different letters are significantly different (*p* < 0.05).

**Table 7:** Digestion model parameters and predicted glycemic index for different breads.

	BB	LYB
C_∞_ (%)	71.17 ± 8.15 ^a^	47.51 ± 3.36 ^b^
K (×10^−2^)	0.0330 ± 0.01 ^a^	0.0467 ± 0.13 ^a^
HI (%)	88.43 ± 0.05 ^a^	62.35 ± 0.03 ^b^
eGI	88.26 ± 0.03 ^a^	73.94 ± 0.02 ^b^

^a^ and ^b^, all values are means of triplicate determinations ± SD. Means with different letters within a row differ significantly (*p* < 0.05).

## Data Availability

Data are contained within the article, further inquiries can be directed to the corresponding authors.
